# Personality Determinants of Diet Quality among Polish and Spanish Physical Education Students

**DOI:** 10.3390/ijerph18020466

**Published:** 2021-01-08

**Authors:** Maria Gacek, Grażyna Kosiba, Agnieszka Wojtowicz

**Affiliations:** 1 Department of Sports Medicine and Human Nutrition, University of Physical Education in Kraków, 31-571 Kraków, Poland; 2 Department of Theory and Methodology of Physical Education, University of Physical Education in Kraków, 31-571 Kraków, Poland; grazyna.kosiba@awf.krakow.pl; 3 Department of Psychology, University of Physical Education in Kraków, 31-571 Kraków, Poland; agnieszka.wojtowicz@awf.krakow.pl

**Keywords:** diet quality indicators, food product groups, physical education students, Big Five personality traits

## Abstract

The aim of the study was to analyze the relationship between the Big Five personality traits and diet quality indexes among Polish and Spanish physical education students. The study was conducted among students from Poland (*n* = 219) and Spain (*n* = 280), using the Dietary Habits and Nutrition Beliefs Questionnaire and the NEO-FFI (NEO Five-Factor Inventory) questionnaire. Two indexes were used: the Pro-healthy Diet Index (pHDI-10) and the Non-healthy Diet Index (nHDI-14). For statistical analysis, the *t*-test with independent estimation of variance as well as both Spearman’s and Pearson’s correlation analysis and moderation analysis with simple slopes were used. Polish and Spanish physical education students demonstrated low levels of healthy (pHDI-10) and unhealthy (nHDI-14) diet indexes, with Polish students showing significantly higher intensities of both indicators (*p* < 0.001). As extraversion intensified, the levels of pHDI-10 and nHDI-14 increased (*p* < 0.05). The nHDI-14 index for all students decreased along with increasing openness to experiences (*p* < 0.01) and agreeableness (*p* < 0.05), and the pHDI-10 index increased with the rise in conscientiousness (*p* < 0.01). Analyses have indicated that the home country is an important moderator of personality relationships with the Non-healthy Diet Index (nHDI-14), which, along with the increase in conscientiousness, increased in students from Spain, while it decreased among students from Poland (*p* < 0.001). Polish and Spanish physical education students showed a low level of healthy (pHDI-10) and unhealthy (nHDI-14) diets depending on country of origin. Additionally, significant correlations were noted between the Big Five personality traits and pHDI-10 and nHDI-14 indexes, and a moderating impact was observed by the home country on the relationships of selected personality traits with the Non-healthy Diet Index (nHDI-14).

## 1. Introduction

A varied and balanced diet, including products with high nutritional density, is one of the most important factors conducive to maintaining health potential and preventing chronic diseases [1]. A rational diet as a key determinant of health has been implemented in the canon of basic aspects of a healthy lifestyle (the WHO pyramid) in many countries including Poland and Spain. Meanwhile, in numerous studies, the prevalence of incorrect food choices has been noted in various population groups, including academic youth with various educational profiles—in Poland [2,3,4], Spain [5,6,7,8,9], as well as other countries [10,11,12].

Due to the fact that nutritional behaviors are determined by numerous psychological and environmental factors [13], an interesting area of research seems to be the assessment of the predictive significance of personality traits included in the Big Five model and the geographical and cultural context in relation to pro-health behaviors, including nutrition. The complex interaction between psychological, cultural, environmental, and behavioral factors may influence the modification of an individual’s eating habits. Earlier studies in the field of personality determinants of nutritional behaviors in academic youth concerned, for example, food choices among students of Polish (Kraków) universities [14,15], eating habits among students of various faculties in Ghana [16], eating disorders in Korean medical students [17] and in Lebanese students [18], eating disorders and alcohol consumption among American students [19], and the intake of dietary supplements by Japanese students [20]. On the other hand, comparative research in the field of geographical and cultural determinants regarding the nutritional behavior of academic youth concerned, among others, students from Spain (Murcia) and Poland (Gdańsk) [21], Spain and Romania [6], and from four other European countries (Poland, Bulgaria, Denmark, and Germany) [22].

In the literature, works may be found regarding the subject of the moderating significance of students’ country of origin with regard to the relationship between personality and physical activity, however, the results are not entirely unambiguous [19,23]. Furthermore, there are no papers that are concerned with a combined analysis of personality and cultural determinants regarding the nutritional choices of academic youth, which was one of the reasons to undertake the research presented in this paper. The authors of works concerning the determinants of students’ pro-health behaviors, including nutrition and level of physical activity, have suggested the legitimacy of further research into the importance of maintaining and improving holistically defined health and the need to clarify the analyzed relationships [16,23].

Within the context of increased physical activity among physical education students and their preparation for their future roles as teachers and health educators of children and youth, research was undertaken on personality determinants regarding the quality of the nutritional choices among students from Poland and Spain assuming the personality and geographical-cultural determinants of nutritional behavior. This work fits into the area of current interdisciplinary research on the determinants of nutritional choices (health creation factors) among academic youth with a field of study focused on physical and health culture. This is also important within the context of American research, in which a positive relationship between rational food choices among teachers and the effectiveness of their nutrition education among schoolchildren has been exhibited [24].

The aim of the study was to analyze the relationship between personality traits included in the Big Five model and diet quality indicators (related to the frequency of consuming selected groups of products with potentially beneficial and adverse effects on health) among Polish and Spanish physical education students, as well as to conduct moderator analysis on the importance of students’ country of origin in relation to the examined correlations.

## 2. Materials and Methods

### 2.1. Survey Design and Sample

Research was conducted in 2017–2019 among 499 s- and third-year 1st-degree (bachelor’s studies) physical education students, aged 18 to 35 (21.65 ± 2.42). The study included 219 Polish students (from the University of Physical Education in Kraków and Wrocław) and 280 Spanish students (from the universities of Murcia and Granada). The study group included 189 women and 310 men. The greatest percentage of Polish students came from urban (35.2%) and rural areas (32.0%); students came less often from medium-sized (19.5%) and small towns (13.3%). A similar percentage of students from Spain came from urban areas (27.1%), small (26.1%), as well as medium-sized cities (24.6%), and from the countryside (22.2%). Questionnaires were conducted in Poland and Spain at the same time (in spring). The trial was carried out by trained persons.

Two standardized tools were used in the study. Food consumption was assessed with a Dietary Habits and Nutrition Beliefs Questionnaire for people aged 15 to 65 (KomPAN), developed by the Committee on Human Nutrition of the Polish Academy of Sciences [25]. The frequency of consuming certain products was assessed on a 6-degree ordinal scale, with the following ranks: 1 (never), 2 (1–3 times a month), 3 (once a week), 4 (several times a week), 5 (once a day), and 6 (several times a day). The original ranks were then converted into real numbers, expressing the daily frequency of food intake (as times/day), according to the formula: never (0), 1–3 times a month (0.06), once a week (0.14), several times a week (0.5), once a day (1) and several times a day (2) [25].

Two diet quality indexes were used: the Pro-healthy Diet Index (pHDI-10) for food intake with potentially beneficial effects on health and the Non-healthy Diet Index (nHDI-14) for the consumption of foods with potentially detrimental effects to health. The pHDI-10 and nHDI-14 indexes were calculated by totaling the daily intake frequency (times/day) of the respective 10 and 14 food groups indicated below. Values of the pHDI-10 index (expressed as daily intake frequency, times/day) were within the range of 0–20, and nHDI-14 values in the range of 0–28. The pHDI-10 values in the range of 0–6.66 are defined as low, within the range of 6.67–13.33 as moderate, and the range of 13.34–20.0 as high. nHDI-14 values between 0–9.33 are defined as low, 9.34–18.66 moderate, and within the range of 18.67–28.0 as high. The interpretation of the indexes is such that the higher their value, the greater the intensity of beneficial or unfavorable features [25].

The Pro-healthy Diet Index (pHDI-10) is related to the frequency of consuming 10 product groups: wholemeal bread, other whole grain cereal products (barley, oatmeal, whole grain pasta, thickly-ground barley), milk, fermented dairy products, fresh cheese, dishes with white meat, fish, legume seeds, fruit, and vegetables. The Non-healthy Diet Index (nHD-14) is determined by the frequency of consuming 14 groups of products: white bread, other purified cereal products (white rice, plain pasta, finely-ground barley), fast food, fried foods, butter, lard, hard and processed cheeses, processed meat products (cold cuts, sausages, hot dogs), red meat dishes, sweets and confectionary, canned meat, sweetened carbonated or non-carbonated beverages, energy drinks, and alcoholic beverages [25].

The NEO-FFI (NEO Five-Factor Inventory) questionnaire, referring to the popular Big Five model (factor analysis that has been applied to personality survey data that revealed semantic associations), was used to assess personality traits [26]. The questionnaire measures the level of five personality traits: neuroticism (sensitive/nervous vs. resilient/confident), extraversion (outgoing/energetic vs. solitary/reserved), openness to experience (inventive/curious vs. consistent/cautious), agreeableness (friendly/compassionate vs. challenging/callous) and conscientiousness (efficient/organized vs. extravagant/careless). NEO-FFI includes a total of 60 self-report items, the truth of which is assessed by the respondent on a five-point scale, in which: 1 means “I strongly disagree”, 2—“I disagree”, 3—“I have no opinion”, 4—“I agree”, 5—“I strongly agree”.

The research was carried out as part of a scientific project entitled “Health behaviors among students of teaching faculties in selected European countries within the context of their future professional role as health educators—analysis of selected formal and legal, socio-cultural and psychological determinants” (University of Physical Education in Kraków, project number: 1136/BS/INS/2017), conducted in accordance with the principles of the 1964 Declaration of Helsinki after obtaining the subjects’ informed consent for participation.

### 2.2. Statistical Analysis

The IBM SPSS 21 program and J.T. Newsom’s macro were used for statistical calculations. Basic statistics of the studied variables were calculated (for diet quality indexes and personality traits: mean and standard deviation; for frequency of consuming selected product groups: median and minimum, maximum). To compare the intensity of personality traits and diet quality indexes (beneficial and unfavorable food choices) between students from Poland and Spain, the *t*-test was used with independent estimation of variance. Spearman’s non-parametric correlation analysis was applied to determine the relationship between the Big Five personality traits and the frequency of consuming selected product groups, while Pearson’s correlation analysis was applied for dietary health quality indexes. To define the differences in relationships between personality traits and the quality of students’ nutritional choices, moderation analysis was used along with simple slopes. The assumed level of statistical significance was *p* = 0.05.

## 3. Results

Among the analyzed personality traits, Polish and Spanish physical education students obtained the highest average results in the areas of extraversion and agreeableness as well as conscientiousness and openness, while lower values were noted for neuroticism (Table 1).

The Polish students were characterized by higher levels of extraversion (*p* < 0.001) as well as conscientiousness (*p* < 0.001), and lower levels of neuroticism (*p* = 0.029) compared to the Spanish students (Table 2).

Analysis of the median value indicates that among products with potentially beneficial effects on health, the students consumed fruits, vegetables, milk, fermented dairy products, and dishes from poultry with the highest average frequency (Me = 0.50); among products with potentially detrimental effects to health, they consumed white rice, finely-ground barley, and processed meat products. Students from Poland consumed butter and yellow cheeses with a higher average frequency, as well as whole grain bread (Table 3).

Analysis of variance indicated that physical education students from Poland demonstrated higher levels of the indicators, both those characteristic for a healthy (*p* = 0.029) and unhealthy diet (*p* < 0.001) compared to students from Spain (Table 4).

In analyzing the relationship between Big Five personality dimensions and the frequency of consuming selected products for the entire studied group of students, it became clear that along with the increase in neuroticism, there was a decrease in the frequency of consuming whole grain cereal products (*p* < 0.05) and lard (*p* < 0.01) as well as sweets and confectionary (*p* < 0.05). With the increase in extraversion, the frequency of consuming fruit, vegetables (*p* < 0.01), whole grains (*p* < 0.05), fried foods, butter (*p* < 0.01), sweets and confectionary (*p* < 0.05), and alcohol (*p* < 0.01) also increased, while the consumption of legume seeds, red meat, canned meat, and lard experienced a decrease (*p* < 0.01). There was also a negative correlation between the level of openness and the frequency of consuming white bread (*p* < 0.01), fried foods, processed meat products, canned meat (*p* < 0.05), butter (*p* < 0.01), sweets as well as confectionary, sweetened beverages (*p* < 0.01), and energy drinks (*p* < 0.05). Along with the increase in agreeableness, the consumption of fast food, fried foods (*p* < 0.05), red meat (*p* < 0.01), processed meat products, as well as sweetened beverages (*p* < 0.05) and energy drinks (*p* < 0.01) decreased. Along with the increase in the level of conscientiousness, the consumption of fruit and vegetables, whole grain cereals, cheese, white meat, fried foods, butter, and sweets as well as confectionary increased, while the consumption of legume seeds, fish, fast food, lard, red and canned meat, and sweetened and energy drinks increased (*p* < 0.01) (Table 5).

With regard to the analyzed diet quality indexes for the whole group, it was shown that as the extraversion intensified, the level of both healthy (pHDI-10) and unhealthy dietary (nHDI-14) choices increased (*p* < 0.05). nHDI-14 decreased with increasing openness to experiences (*p* < 0.01) and agreeableness (*p* < 0.05), while pHDI-10 increased with the intensification of conscientiousness (*p* < 0.01) (Table 5).

Moderation analyses have shown that home country is not a statistically significant moderator of personality relationships with the Pro-healthy Diet Index (pHDI-10) among physical education students from Poland or Spain (Table 6).

**Figure 1:**
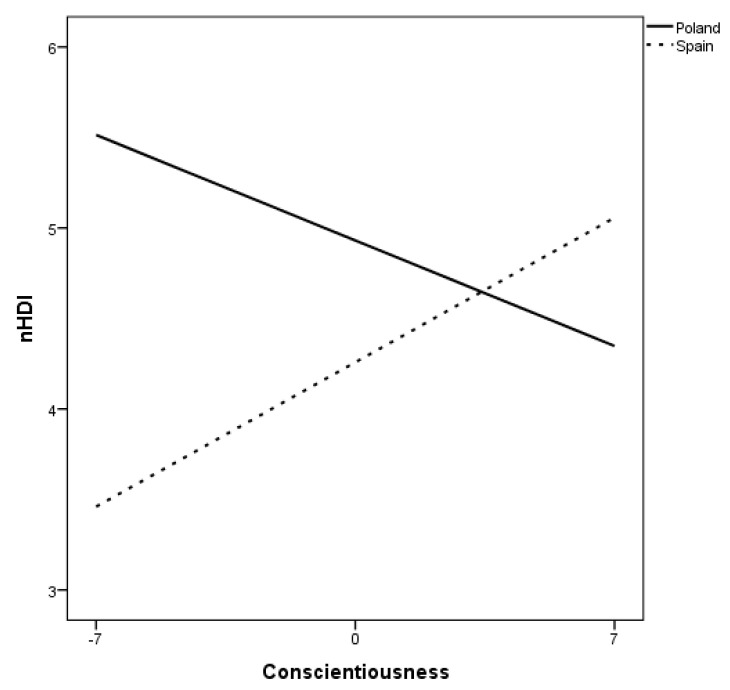
Relationship between Non-healthy Diet Index (nHDI-14) and the level of conscientiousness according to home country among Polish and Spanish physical education students.

Moderation analyses have indicated that country of origin is a statistically significant moderator of the relationship between the Non-healthy Diet Index (nHDI-14) and the personality of the surveyed physical education students. It has been shown that the correlation between conscientiousness and the level of unhealthy food choices differs among students from Poland and Spain (*p* < 0.001). Along with the intensification of conscientiousness, the Non-healthy Diet Index (nHDI-14) among students from Spain increased, while it decreased for students from Poland (Table 7, Figure 1).

## 4. Discussion

Among Polish and Spanish physical education students, differentiation of personality traits and dietary health quality indicators were demonstrated. This also applies to the relationships between Big Five personality dimensions and the consumption of certain product groups and dietary quality indicators, and furthermore, the moderating influence of the home country on the relationships between certain personality dimensions and the quality of food choices.

The examined physical education students showed different intensities of individual personality traits, with extraversion being the highest intensity and neuroticism the lowest. Polish students were characterized by a higher intensity of extraversion and a lower intensity of neuroticism than students from Spain. Such a configuration of personality traits may be associated with the field of study, because physical activity may realize the high demand for stimulation (with high extraversion) and improve emotional state. The relatively high level of openness and conscientiousness of the surveyed physical education students corresponds to the results of research obtained for students of various university faculties from Ghana [16].

The indicated nutritional mistakes of the entire studied group of students were particularly associated with the low frequency of consuming fruit and vegetables, whole grain cereals, and fermented dairy products recommended in the daily diet, as well as the relatively frequent consumption of non-recommended meat products (cold cuts, sausages, hot dogs). As expected, Polish students more often consumed butter and cheese, which are products not recommended in the Mediterranean diet due to their high content of saturated fatty acids. Assessment of the average daily frequency of consuming products with potentially beneficial as well as those with potentially adverse effects on health also allowed the determination of the levels of healthy (pHDI-10) and unhealthy (nHDI-14) indicators, the values of which oscillated at a low level, which means weak choices in both beneficial as well as unfavorable food choices and thereby weak showings in both protective and anti-health effects of the diet. Insufficient consumption of products with high nutritional value may have reduced the supply of various nutrients, including dietary fiber (vegetables, fruit, whole grains), potassium and magnesium (vegetables), as well as antioxidant vitamins and polyphenols (vegetables, fruits). The infrequent consumption of fermented dairy products may have reduced the supply of probiotics with numerous pro-health values. On the other hand, relatively frequent consumption of processed meat products could have increased the supply of saturated fatty acids and cholesterol exhibiting excessive hyperlipidemic and hypertensive properties [1].

In other studies, nutritional abnormalities were confirmed among academic youth, including physical education students, both in Poland [2,4] and Spain [6], as well as Chile [27]. Incorrect nutritional decisions were described among other university faculties, including Polish [3,28,29] and Spanish students [5,7,8,9]. The nutritional tendencies observed in the study discussed by the author are related to the results of research in another group of Polish physical education students (from Biała Podlaska), in which it was shown that students did not often choose products with potentially beneficial effects on health, but at the same time, they limited the consumption of unhealthy products [2]. Similarly in the case of Chilean physical education students, low consumption of vegetables and dairy products was noted among men, as well as excessive consumption of sweet snacks among women, which also reduced the health values of the diet [27].

The discussed research among Polish and Spanish physical education students also showed statistically significant differences in the quality of food choices depending on country of origin, with an indication of a higher rate of both favorable and unfavorable food choices among Polish students. In other studies among Polish (from Gdańsk) and Spanish (from Murcia) physical education students, it was also confirmed that there is diversity in eating behaviors depending on country, with students from Murcia more often consuming seafood and dairy products, while students from Gdańsk consumed more vegetables [21]. Diversification of eating behavior depending on the geographical factor was also shown in research conducted among physical education students from Spain and Romania. In this study, it was observed that Spanish students were more likely to follow the recommendations of the Mediterranean diet than Romanian students [6].

In the discussed studies, statistically significant correlations were also shown between the Big Five personality traits and the frequency of consuming selected food groups as well as the Pro-healthy (pHDI-10) and the Non-healthy Diet Index (nHDI-14). It was found that along with the increase in the level of neuroticism associated with emotional lability, low self-esteem, and high sensitivity, the consumption of recommended whole grain cereals clearly decreased, while the consumption of non-recommended products (including sweets) increased. With the intensification of extraversion, associated with assertiveness, optimism, and the search for sensations, consumption of the majority of recommended products increased, while the amount of non-recommended products experienced a decrease (including red meat and lard). However, the consumption of some non-recommended products also increased (sweets and alcohol). It was also shown that the increase in openness and agreeableness caused a rise in the consumption of non-recommended products. In turn, along with the increase in conscientiousness, associated with the obligation and pursuit of specific goals, the consumption of the majority of recommended products increased, while the consumption of most of the non-recommended products decreased. In the case of the Pro-healthy Diet Index (pHDI-10), a high level of conscientiousness was important, and for the smaller scale of Non-healthy Diet Index (nHDI-14), so was a high level of openness and agreeableness. Extraversion was associated with both potentially beneficial (pHDI-10) and detrimental (nHDI-14) food choices. Recalling the personality characteristics discussed above, it should be noted that the surveyed students were characterized by a high level of extraversion, agreeableness, conscientiousness and openness, and a lower level of neuroticism, which is rather a positive psychological basis for making health-promoting food choices and limiting products adverse to health.

The demonstrated regularities are explained in the characteristics of the analyzed personality dimensions, and also refer to the results of studies by other authors on the psychological determinants of nutritional behavior among academic youth. Among university students in Ghana, relationships between Big Five personality traits (in addition to neuroticism) and nutritional behaviors were demonstrated in such a way that extraversion and openness were positively associated with nutritional interests, conscientiousness with diet diversity and limiting sugar intake, and agreeableness with skipping meals, neophagy and diet variety [16]. Relationships of extraversion with more frequent fish consumption and low neuroticism and regularity of meals were demonstrated among students of Kraków universities undertaking different fields of study [14]. On the other hand, among Japanese students, the positive relationship between extraversion and the use of dietary supplements was confirmed [20]. Extraversion and conscientiousness were strong predictors of pro-health behaviors among American students, indicating that students with high levels of conscientiousness had healthier lifestyles; in eating behaviors, students with high levels of this indicator consumed fruit and vegetables significantly more often than students with a low level [30]. This corresponds to the results of the authors’ research, because the Pro-healthy Diet Index (pHDI-10) includes, among others, consumption of fruit and vegetables, which should form the basis of a daily diet. In other studies, the relationship between conscientiousness and rational eating behaviors was confirmed, in particular, in limiting the consumption of sweets and preferring fruit in one’s diet [31,32]; openness to advice was related to healthy dietary practices [33,34] and controlling the supply of fats (unlike extraversion) [35]. The relationship between neuroticism and less favorable nutritional choices of physical education students demonstrated in the authors’ research was also confirmed by studies conducted by other authors [31,32].

The moderating influence of the country of origin on the relationships between some of the Big Five personality dimensions and the health quality of the diet described in the discussed research was such that the country did not affect personality relationships with the Pro-healthy Diet Index (pHDI-10), but moderated the relationship of personality with the Non-healthy Diet Index (nHDI-14). It has been shown that the relationship of conscientiousness with the level of unfavorable food choices was different among students from Poland and Spain, because while among students from Poland this indicator decreased with the increase in conscientiousness, it increased in students from Spain (differently than for the whole group).

The issue of the moderating importance of the home country in relation to the correlation between personality traits and pro-health behaviors is poorly explored. In the literature, works have been presented on the determinants of physical activity, which, however, did not allow clear identification of the moderators of the relationship between personality and physical activity [23]. In other studies on the health behaviors of physical education students from Gdańsk and Murcia, it was confirmed that there is diversity in certain aspects of lifestyle, with Spanish students showing more rational dietary choices [21]; this is not unequivocally confirmed in the authors’ research. The authors of research on the relationship between satisfaction with body mass, rational nutrition, health awareness and physical activity of students from various cultures (Egypt, Palestine, and Finland) [36] also suggested the justification for continuing research in various countries.

The research discussed pointed to the predictive significance of personality traits and geographical context (country of origin) in relation to the quality of food choices among Polish and Spanish physical education students. Diagnosing individual determinants of eating behaviors as important health resources may increase the efficiency of the students’ diet rationalization. At the same time, it is necessary to point out the legitimacy of further research on the psychological determinants of the nutritional behavior of academic youth, taking into account the larger size and diversity of the group and the broader spectrum of psychological and nutritional analyses, which correspond to the suggestions of other authors [16].

In conclusion, the limitations and strengths of the study, as indicated, are mainly related to the fact that the applied nutritional questionnaire (KomPAN) is a Polish tool that does not fully take the specificity of the Mediterranean diet into account (although it contains the main recommended and non-recommended products in this diet). Furthermore, it was not validated for the Spanish population. In order to obtain more unambiguous results in future studies, it is possible to use a modified questionnaire regarding the consumption of food groups, in accordance with the specificity of the Mediterranean diet, and additionally, to extend the analysis as to quantify the diet. Limitations of the work are also related to the questionnaire research (self-report tools), hence, the collected data are declarative. There are also limitations related to the failure to consider socio-economic factors. Future research, taking into account a wide range of individual and environmental factors, could contribute to the development of a complex model of food choice determinants. The strengths of our study are its interdisciplinary nature (nutritional and psychological aspects) and the selection of a group of students from different cultures who are preparing to implement school health education, an important area of which is nutritional education.

## 5. Conclusions

1. Among Polish and Spanish physical education students, improper dietary choices as well as low levels of healthy (pHDI-10) and unhealthy (nHDI-14) diet indexes have been shown. Significant differences in diet quality depended on the country of origin, with higher pHDI-10 and nHDI-14 values being indicated among students from Poland.

2. Significant correlations between the Big Five personality traits and the frequency of consuming selected food groups, as well as pHDI-10 and nHDI-14 indexes, were found among Polish and Spanish physical education students. The high level of healthy diets was associated with higher levels of conscientiousness, and the lower level of unhealthy diets with a high level of openness and agreeableness. Extraversion was associated with both healthy and unhealthy nutritional choices.

3. Among Polish and Spanish physical education students, the moderate influence of the country of origin on the relationships of selected Big Five personality traits with the Non-healthy Diet Index (nHDI-14) was demonstrated, since along with the increase in conscientiousness, the level of unhealthy food choices among Polish students decreased, while for students from Spain, this level experienced an increase.

## Figures and Tables

**Table 1:** Intensity of Big Five personality traits for the total number of Polish and Spanish physical education students (descriptive statistics).

Variables		*n*	Descriptive Statistics	
			M	SD
Personality traits (NEO-FFI)	Neuroticism	499	33.34	6.26
	Extraversion	499	40.48	5.71
	Openness	499	37.15	4.36
	Agreeableness	499	39.69	4.58
	Conscientiousness	499	37.89	7.11

Legend: M—arithmetic mean, SD—standard deviation.

**Table 2:** Comparison of the intensity of Big Five personality traits among physical education students from Poland (*n* = 219) and Spain (*n* = 280).

Variables		M Poland	M Spain	*p*	SD Poland	SD Spain
NEO-FFI	Neuroticism	32.58	33.93	0.029 *	8.57	3.43
	Extraversion	43.34	38.23	<0.001 *	6.04	4.26
	Openness	36.92	37.34	0.323 *	5.48	3.22
	Agreeableness	39.84	39.58	0.534 *	5.52	3.68
	Conscientiousness	43.79	33.27	<0.001 *	6.46	2.98

Legend: *—test with independent estimate of variance, M—arithmetic mean, SD—standard deviation, *p*—statistical significance.

**Table 3:** Daily frequency of consuming products with potentially beneficial and adverse effects on health and the values of the Pro-healthy (pHDI-10) and the Non-healthy Diet Index (nHDI-14) among the examined Polish and Spanish physical education students (*n* = 499) (descriptive statistics).

Evaluated Categories (Groups of Food Products and Indexes of Diet Quality)		Me All	Me Poland	Me Spain	Min	Max
Potentially beneficial influence on health (pHDI-10)	Fruit	0.50	0.50	0.50	0	2.00
	Vegetables	0.50	0.50	0.50	0	2.00
	Wholemeal bread	0.14	0.50	0.06	0	2.00
	Thickly-ground barley, oatmeal, whole wheat pasta	0.14	0.50	0.14	0	2.00
	Legume seeds	0.14	0.14	0.14	0	2.00
	Milk	0.50	0.50	0.50	0	2.00
	Fermented dairy products	0.50	0.50	0.50	0	2.00
	Fresh cheese	0.14	0.14	0.06	0	2.00
	Poultry dishes	0.50	0.50	0.50	0	2.00
	Fish dishes	0.14	0.14	0.14	0	2.00
Potentially detrimental influence on health (nHDI-14)	White bread	0.50	0.50	0.14	0	2.00
	White rice, finely-ground barley	0.50	0.50	0.50	0	2.00
	Hard, soft, or processed cheeses	0.14	0.50	0.06	0	2.00
	Cold cuts, sausages, hot dogs	0.50	0.50	0.50	0	2.00
	Dishes from red meat	0.14	0.14	0.14	0	2.00
	Canned meat	0.06	0.06	0.06	0	1.00
	Fried foods (farinaceous and meat)	0.14	0.50	0.14	0	2.00
	Butter	0.06	0.50	0.06	0	2.00
	Lard	0.06	0.00	0.06	0	2.00
	Fast food	0.06	0.06	0.06	0	2.00
	Sweets and confectionary	0.14	0.50	0.14	0	2.00
	Sweetened carbonated and non-carbonated drinks	0.06	0.06	0.06	0	2.00
	Energy drinks	0.06	0.06	0.06	0	2.00
	Alcoholic beverages	0.06	0.14	0.06	0	2.00

Legend: Me—median.

**Table 4:** Comparison of the Pro-healthy (pHDI-10) and the Non-healthy Diet Index (nHDI-14) among physical education students from Poland (*n* = 219) and Spain (*n* = 280) (*t*-test).

Index	M All	M Poland	M Spain	*p*	SD All	SD Poland	SD Spain
pHDI-10	4.65	4.92	4.43	0.029 *	2.57	2.27	2.76
nHDI-14	4.05	4.45	3.74	<0.001	2.60	2.27	2.02

Legend: *—*t*-test with independent estimation of variance, M—arithmetic mean, SD—standard deviation, *p*—statistical significance.

**Table 5:** Relationships between the Big Five personality traits and the frequency of consuming foods with potentially beneficial and adverse effects on health and the values of diet quality indexes (pHDI-10 and nHDI-14) among the total number of examined Polish and Spanish physical education students (*n* = 499) (Spearman’s Rho and Pearson’s r).

Evaluated Categories (Groups of Food Products and Indexes of Diet Quality)		Spearman’s Rho				
		N	E	O	A	C
Potentially beneficial (pHDI-10)	Fruit	−0.01	0.16 **	0.07	0.09	0.14 **
	Vegetables	−0.05	0.21 **	0.08	0.06	0.29 **
	Wholemeal bread	0.03	0.12 **	−0.03	0.05	0.21 **
	Thickly-ground barley, oatmeal, whole-wheat pasta	−0.09 *	0.09 *	0.07	0.06	0.14 **
	Legume seeds	0.03	−0.09 **	0.08	0.07	−0.23 **
	Milk	0.07	−0.05	−0.02	−0.03	0.05
	Fermented dairy products	0.07	−0.02	−0.07	0.00	0.06
	Fresh cheese	−0.02	−0.02	−0.03	−0.07	0.22 **
	Poultry dishes	−0.06	0.09	−0.04	−0.08	0.12 **
	Fish dishes	0.06	−0.06	0.04	−0.03	−0.13 **
Potentially detrimental (nHDI-14)	White bread	−0.03	0.03	−0.17 **	−0.00	0.05
	White rice, finely-ground barley	−0.04	0.06	0.02	−0.04	0.09
	Hard, soft, and processed cheeses	−0.04	0.09	−0.04	0.05	0.22 **
	Cold cuts, sausages, hot dogs	0.02	0.01	−0.10 *	−0.10 *	0.01
	Dishes from red meat	0.01	−0.12 **	0.04	−0.16 **	−0.13 **
	Canned meat	0.06	−0.15 **	−0.10 *	−0.06	−0.27 **
	Fried foods (farinaceous and meat)	−0.04	0.15 **	−0.11 *	−0.09 *	0.22 **
	Butter	0.05	0.13 **	−0.14 **	0.02	0.13 **
	Lard	0.13 **	−0.19 **	−0.06	−0.03	−0.36 **
	Fast food	0.04	−0.06	−0.07	−0.10 *	−0.16 **
	Sweets and confectionary	0.09 *	0.09 *	−0.17 **	0.00	0.21 **
	Sweetened carbonated and non-carbonated beverages	−0.01	−0.02	−0.15 **	−0.10 *	−0.15 **
	Energy drinks	0.01	−0.03	−0.10 *	−0.12 **	−0.15 **
	Alcoholic beverages	−0.09	0.21 **	0.06	−0.00	0.08
		Pearson’s r				
Indexes	pHDI-10	−0.02	0.09 *	0.08	0.06	0.14 **
	nHDI-14	−0.03	0.09 *	−0.13 **	−0.11 *	0.06

Legend: N—neuroticism, E—extraversion, O—openness, A—agreeableness, C—conscientiousness, *—*p* < 0.05, **—*p* < 0.01 (two-sided statistically significant correlations).

**Table 6:** Moderation analysis—moderating variable: country; dependent variable: pHDI-10.

Dep. Variable	Moderator	Ind. Variable	β	SE	t	*p* Interaction	Slopes
Pro-healthy diet indicator (pHDI-10)	Country	Neuroticism	0.05	0.05	0.94	0.350	β _P_ = −0.03 (*p* = 0.609) β _S_ = 0.09 (*p* = 0.427)
		Extraversion	−0.05	0.06	−0.76	0.450	β _P_ = 0.10 (*p* = 0.130) β _S_ = 0.02 (*p* = 0.808)
		Openness	0.01	0.05	0.18	0.858	β _P_ = 0.08 (*p* = 0.154) β _S_ = 0.09 (*p* = 0.245)
		Agreeableness	−0.10	0.06	−1.86	0.063	β _P_ = 0.12 (*p* = 0.032) β _S_ = −0.05 *p* = 0.479)
		Conscientiousness	−0.07	0.07	−1.00	0.318	β _P_ = 0.19 (*p* = 0.010) β _S_ = 0.03 (*p* = 0.823)

Legend: Dep. Variable—dependent variable, Ind. Variable—independent variable, β—standardized Beta coefficient, SE—standard error, *p*—statistical significance, P—Poland, S—Spain.

**Table 7:** Moderation analysis–moderating variable: country; dependent variable: nHDI-14.

Dep. Variable	Moderator	Ind. Variable	β	SE	t	*p* Interaction	Slopes
Non-healthy diet indicator (nHDI-14)	Country	Neuroticism	0.05	0.05	1.08	0.282	β _P_ = −0.04 (*p* = 0.449) β _S_ = 0.09 (*p* = 0.401)
		Extraversion	0.06	0.06	0.97	0.331	β _P_ = −0.02 (*p* = 0.790) β _S_ = 0.08 (*p* = 0.303)
		Openness	0.09	0.05	1.76	0.078	β _P_ = −0.17 (*p* = 0.001) β _S_ < 0.001 (*p* = 0.962)
		Agreeableness	0.08	0.05	1.56	0.119	β _P_ = −0.17 (*p* = 0.003) β _S_ = −0.02 (*p* = 0.740)
		Conscientiousness	0.29	0.07	4.09	<0.001	β _P_ = −0.27 (*p* < 0.001) β _S_ = 0.37 (*p* = 0.008)

Legend: Dep. variable—dependent variable, Ind. variable—independent variable, β—standardized Beta coefficient, SE—standard error, *p*—statistical significance, P—Poland, S—Spain.

## Data Availability

Data available on request due to restrictions eg privacy or ethical.
